# Neuroplastinβ-mediated upregulation of solute carrier family 22 member 18 antisense (SLC22A18AS) plays a crucial role in the epithelial-mesenchymal transition, leading to lung cancer cells' enhanced motility

**DOI:** 10.1016/j.bbrep.2020.100768

**Published:** 2020-05-17

**Authors:** Karolina Bajkowska, I. Wayan Sumardika, Nahoko Tomonobu, Youyi Chen, Ken-ichi Yamamoto, Rie Kinoshita, Hitoshi Murata, Ni Luh Gede Yoni Komalasari, Fan Jiang, Akira Yamauchi, I. Made Winarsa Ruma, Carlos Ichiro Kasano-Camones, Yusuke Inoue, Masakiyo Sakaguchi

**Affiliations:** a Department of Cell Biology, Okayama University Graduate School of Medicine, Dentistry and Pharmaceutical Sciences, 2-5-1 Shikata-cho, Kita-ku, Okayama-shi, Okayama 700-8558, Japan; b University of Surrey, 11 Osterley Court, London TW7 4PX, England, UK; c Faculty of Medicine, Udayana University, Denpasar 80232, Bali, Indonesia; d Department of General Surgery & Bio-Bank of General Surgery, The Fourth Affiliated Hospital of Harbin Medical University, Harbin, 150001, China; e Department of Biochemistry, Kawasaki Medical School, 577 Matsushima, Kurashiki-shi, Okayama 701-0192, Japan; f Faculty of Science and Technology, Division of Molecular Science, Gunma University, 1-5-1 Tenjin-cho, Kiryu-shi, Gunma 376-8515, Japan

**Keywords:** Lung cancer, Metastasis, Epithelial-mesenchymal transition, Solute carrier family 22 member 18 antisense, S100A8/A9, Neuroplastin

## Abstract

Our recent study revealed an important role of the neuroplastin (NPTN)β downstream signal in lung cancer dissemination in the lung. The molecular mechanism of the signal pathway downstream of NPTNβ is a serial activation of the key molecules we identified: tumor necrosis factor (TNF) receptor-associated factor 2 (TRAF2) adaptor, nuclear factor (NF)IA/NFIB heterodimer transcription factor, and SAM pointed-domain containing ETS transcription factor (SPDEF). The question of how dissemination is controlled by SPDEF under the activated NPTNβ has not been answered. Here, we show that the NPTNβ-SPDEF-mediated induction of solute carrier family 22 member 18 antisense (SLC22A18AS) is definitely required for the epithelial-mesenchymal transition (EMT) through the NPTNβ pathway in lung cancer cells. *In vitro*, the induced EMT is linked to the acquisition of active cellular motility but not growth, and this is correlated with highly disseminative tumor progression *in vivo*. The publicly available data also show the poor survival of SLC22A18AS-overexpressing lung cancer patients. Taken together, these data highlight a crucial role of SLC22A18AS in lung cancer dissemination, which provides novel input of this molecule to the signal cascade of NPTNβ. Our findings contribute to a better understanding of NPTNβ-mediated lung cancer metastasis.

## Highlights

• SLC22A18AS is upregulated by NPTNβ activation.
• SLC22A18AS plays a crucial role in the cancer epithelial-mesenchymal transition.

## Abbreviations

ALCAM: activated leukocyte cell adhesion molecule
CDH: cadherin
EMMPRIN: extracellular matrix metalloproteinase inducer
EMT: epithelial-mesenchymal transition
FGFR4: fibroblast growth factor receptor 4
GRB2: growth factor receptor-bound protein 2
IGFBP2: insulin-like growth factor binding protein 2
ITGA1: integrin subunit alpha 1
ITGB4: integrin subunit beta 4
KLF5: kruppel-like factor 5
MCAM: melanoma cell adhesion molecule
NF: nuclear factor
NPTN: neuroplastin
RAGE: receptor for advanced glycation endproducts
SLC22A18AS: solute carrier family 22 member 18 antisense
SNAIL: snail family transcriptional repressor
SPDEF: SAM pointed-domain containing ETS transcription factor
TLR4: toll-like receptor 4
TNF: tumor necrosis factor
TRAF2: TNF receptor-associated factor 2
VIM: vimentin
ZEB: zinc finger E-box binding homeobox

## 1 Introduction

Cancer metastasis is a grave problem for cancer patients because of its life-threatening nature, which may involve multiple-organ dysfunction due to the disseminated cancer’ growth. To overcome the problem of the ability of cancer cells to metastasize, a mechanistic understanding of cancer metastasis is necessary. The ‘seed and soil’ theory that was first proposed by Paget et al., in 1989 [1] may provide critical clues regarding the complex mechanism of distant cancer metastasis. In that theory, the term ‘seed’ indicates the cancer cells and ‘soil’ represents the favored organ of the cancer for the destination of metastasis. The seed and soil theory has not been solved at the molecular level. Hiratsuka et al. made a breakthrough in 2006–2008 by identifying the relationship between the heterodimic calcium-binding protein S100A8/A9 and Toll-like receptor 4 (TLR4) [2,3].

A major metastatic destination of cancer is the lung. The lung senses distant cancer cells and then secretes an abundant amount of S100A8/A9, which in turn attracts distant cancer cells through their surface TLR4. Saha et al. demonstrated that receptor for advanced glycation end-products (RAGE) on the surface of cancer cells also functions as an S100A8/A9-sensing receptor [4]. In addition to TLR4 and RAGE, we succeeded in identifying four novel S100A8/A9 receptors: melanoma cell adhesion molecule (MCAM), activated leukocyte cell adhesion molecule (ALCAM) [5], extracellular matrix metalloproteinase inducer (EMMPRIN) [6], and neuroplastin (NPTN)β (Suppl. Fig. S1) [7]. These receptors seem to be differently expressed depending on the cancer species, and their expression seems to be upregulated in response to the progression of cancer malignancy.

Among these receptors, we reported a crucial role of an S00A8/A9-NPTNβ axis in the progression of the dissemination of lung cancer *in vitro* and *in vivo* [8] (Suppl. Fig. S2). Our homology research identified NPTNβ as one of the novel S100A8/A9 receptors on the basis of the cytoplasmic tail of EMMPRIN [7]. We later observed that the relationship between EMMPRIN and NPTNβ is a paralog. NPTNβ is a type I transmembrane protein that belongs to the Ig superfamily (Suppl. Fig. S1); it is highly expressed in neuronal cells [9] and has functions in cell adhesion [10] and the promotion of synaptic activity [11] and plasticity [12]. NPTNβ is also expressed at a significant level in lung cancer cells, and NPTNβ plays a crucial role in its disseminative progression when it functions as a receptor to the extracellular S100A8/A9 [8]. What downstream signal(s) does this axis use as a driving force for cancer progression in the lung? We discovered a key transcription factor, nuclear factor (NF)IA/NFIB, which is positively regulated mostly by tumor necrosis factor (TNF) receptor-associated factor 2 (TRAF2), but the growth factor receptor bound protein 2 (GRB2)-RAS pathway also contributes to the activation of NFIA/NFIB in an orchestrated manner with TRAF2. The activation of NFIA/NFIB then leads to an induction of SAM pointed domain containing ETS (SPDEF) transcription factor, which greatly contributes to lung cancer progression with disseminative activities (Suppl. Fig. S2).

Taken together, our data support the notion that the newly identified NPTNβ signaling pathway that is initiated by cancer surrounding extracellular S100A8/A9 worsens the disseminating progression of lung cancers. However, it remained to be addressed how an S100A8/A9-NPTNβ signal that led to SPDEF activation might control the upregulation of lung cancer dissemination. We conducted the present study to investigate this in the context of lung cancer, toward the goal of uncovering the signal pathway of NPTNβ.

## 2 Materials and methods

### 2.1 Cell lines

The cell lines used were as follows: HEK293T (a human embryonic kidney cell line stably expressing the SV40 large T antigen; RIKEN BioResource Center, Tsukuba, Japan) and A549 (a human lung adenocarcinoma cell line with *KRAS*-mutant [G12S]; ATCC, Rockville, MD). These cells were all cultivated in D/F medium (ThermoFisher Scientific, Waltham, MA) supplemented with 10% fetal bovine serum (FBS).

### 2.2 RNA-seq and quantitative real-time PCR

Total RNA was extracted from the cells with ISOGEN (Nippon Gene, Tokyo) according to the manufacturer's instructions. The isolated RNA was subjected to an RNA-seq-based analysis of gene expression (Bioengineering Lab, Kanagawa, Japan). A real-time reverse transcription-polymerase chain reaction (RT-PCR) was performed in a LightCycler rapid thermal cycler system (Roche Diagnostics, Indianapolis, IN) using a LightCycler 480 SYBR Green I Master Kit (Roche Diagnostics) according to the manufacturer's instructions.

The forward and reverse primer pairs used (5′ to 3′) were as follows: *FGFR4* (forward: gccgtcaagatgctcaaag [Tm = 58 °C], reverse: gatcagcttcatcacctccat [Tm = 59 °C]), *IGFBP2* (forward: ccaagaagctgcgaccac [Tm = 60 °C], reverse: ggagtagaggtgctccagagg [Tm = 62 °C]), *ITGA1* (forward: cacatgtaaagttggatatcccttc [Tm = 59 °C], reverse: ggtcacattttccatgagatagg [Tm = 58 °C]), *ITGB4* (forward: ggagtaccagctgctgaacg [Tm = 62 °C], reverse: cggaacacgtaggagtggtt [Tm = 61 °C]), *KLF5* (forward: cgcatccactactgcgatta [Tm = 59 °C], reverse: tgtgagtcctcaggtgagctt [Tm = 62 °C]), *SLC22A18AS* (forward: gcttggtggttctctcctgat [Tm = 61 °C], reverse: agtccttctgcgccctct [Tm = 62 °C]), *SLC22A18* (Forward: catcttgcttacctacgtgctg [Tm = 60 °C], reverse: cccagtttccgagacaggta [Tm = 60 °C]), *CDH1* (forward: cttactgcccccagaggat [Tm = 60 °C], reverse: gctggctcaagtcaaagtcc [Tm = 60 °C]), *CDH2* (forward: aatggatgaaagacccatccac [Tm = 60 °C], reverse: gagccactgccttcatagtcaa [Tm = 61 °C]), *VIM* (forward: gaccagctaaccaacgacaaa [Tm = 60 °C], Reverse: gaagcatctcctcctgcaat [Tm = 59 °C]), *ZEB1* (forward: ggaggatgacacaggaaagg [Tm = 59 °C], reverse: tctgcatctgactcgcattc [Tm = 59 °C]), *ZEB2* (forward: aggagctgtctcgccttg [Tm = 60 °C], Reverse: ggcaaaagcatctggagttc [Tm = 58 °C]), *SNAIL1* (forward: gctgcaggactctaatccaga [Tm = 60 °C], reverse: atctccggaggtgggatg [Tm = 59 °C]), *SNAIL2* (forward: acagcgaactggacacacat [Tm = 61 °C], reverse: gatggggctgtatgctcct [Tm = 60 °C]), *TBP* (forward: gaacatcatggatcagaacaaca [Tm = 58 °C], reverse: atagggattccgggagtcat [Tm = 59 °C]). The *TBP* gene expression was used as a reliable calibration control.

### 2.3 Plasmids and A549 cell-originated clones

**Fig. 1:**
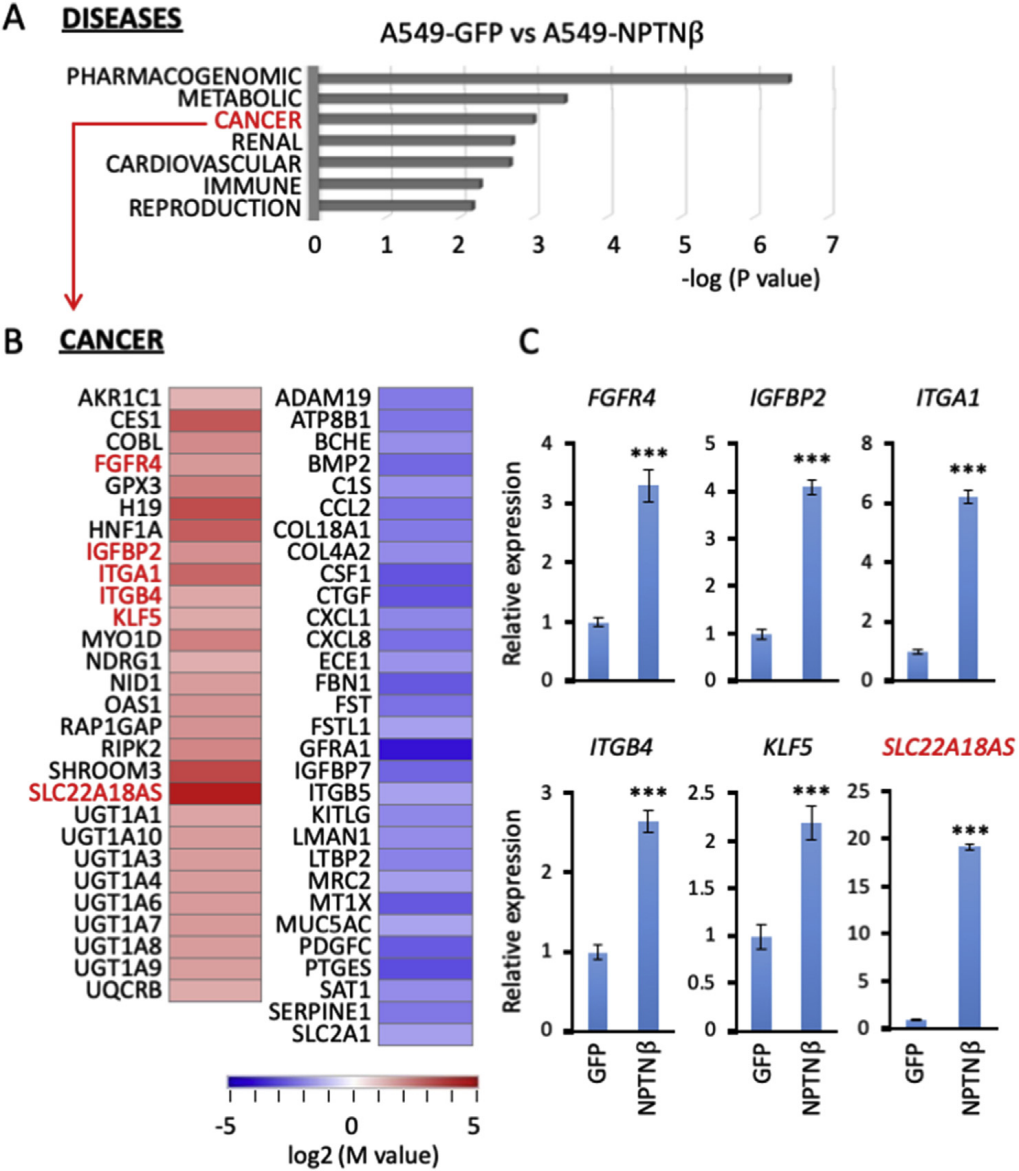
**RNA-seq based analysis. A:** A functional enrichment analysis (p<0.05) was performed for protein-coding RNAs in GAD_DISEASE_CLASS with upregulated and downregulated genes in the A549-derived NPTNβ-overexpressing clone in a comparison with those in the GFP-overexpressing clone. **B:** Heat map for the selected genes that are upregulated and downregulated in the cancer category of the disease clustering as indicated in panel (A). The analysis was performed using the database for annotation, visualization and integrated discovery (DAVID) v6.8 (http://david.ncifcr.gov/). **C:** A quantitative real-time PCR analysis was carried out in the indicated cells on the x-axis for the genes checked in red in panel (B). The relative expression level of each sample is shown after calibration with TBP gene (a suitable housekeeping gene) value. Data are mean ± SD. ***p<0.001. (For interpretation of the references to color in this figure legend, the reader is referred to the Web version of this article.)

The pIDT-SMART (C-TSC) vector, abbreviated as pCMViR-TSC [13], was used for the transient expression of foreign genes. The cDNAs of green fluorescence protein (GFP), human SLC22A18 sense, and its reverse sequence as SLC22A18AS were inserted into the multi-cloning site of the pCMViR-TSC vector. The cells were transiently transfected with the plasmid vectors using FuGENE-HD (Promega, Madison, WI). We established A549-originated clones (A549-GFP and A549-NPTNβ, Fig. 1) that permanently express control GFP and NPTNβ [8] and clones (AS#1 and AS#3) that express SLC22A18AS in a stable manner by a convenient electroporation gene delivery method using pSAKA-4B [8,14,15] and subsequent selection with puromycin at 20 μg/ml.

### 2.4 Cell growth and motility assays

We used the CellTiter 96® AQueous One Solution Cell Proliferation Assay (MTS) (Promega) for the assessment of cell proliferation. Migration and invasion assays were performed by the Boyden chamber method. For the invasion assay, 8-μm-pore filters set in Transwell culture inserts (BD Biosciences) were coated with Matrigel. Cells (3 × 10^4^ cells/insert) were placed in the upper chamber in a low-serum medium, D/F (0.5% FBS), and the lower chamber was filled with a high-serum medium, D/F (10% FBS). Twelve hr later, the cells that had passed through the membrane were stained with hematoxylin and eosin (H&E) (Muto Pure Chemicals, Tokyo). Each Transwell insert was microscopically imaged in five distinct regions at 100 × magnification in triplicate. The numbers of cells that had migrated to the five non-overlapping regions were counted using BZ-analysis software (Keyence, Osaka, Japan) and summed as the total cell number and are presented as the average of three independent experiments.

### 2.5 *In vivo* mouse study

Seven-week-old female nude mice, i.e., BALB/c-nu/nu mice, were purchased from CLEA Japan (Tokyo). All mice were provided with sterilized food and water and were housed in a barrier facility under a 12:12-hr light/dark cycle. A549 cells and their derivative clone AS#3 (5 × 10^5^ cells with Matrigel/50 μl/mouse) were directly injected with a syringe (30-gauge needle) into the lungs of three mice per one group in total two groups (3 mice/A549 cells; 3 mice/clone AS#3 cells), respectively, under isoflurane anesthesia. The needle was inserted approx. 0.5 cm deep from the surface of the mouse's chest. One important parameter to judge the successful injection to the inside of a mouse lung is the feeling of slight pressure during the injection procedure. Isoflurane was used at concentrations of 4%–5% for the induction and 2%–3% for the maintenance of anesthesia. At 1 month after the injection, the whole lungs were isolated and observed macroscopically, and the disseminated cell-derived tumor foci >1 mm in dia. were counted.

### 2.6 Image acquisition

Phase contrast images of the cultured cells were acquired using BZ-X700 all-in-one fluorescence microscope (Keyence). Imaging mode, phase contrast (focal distance 35 mm); magnification, x 200; pixel resolution, 960 × 720. The dissected mouse lung images were captured using an iPhone 7 (Apple, Cupertino, CA). Imaging mode, digital camera; magnification, x 1; pixel resolution, 4032 × 3024. The acquired images were then edited using Microsoft Suite software (cropped, contrast +0, brightness +20).

### 2.7 Statistical analyses

All values are expressed as the mean ± SD. All data were analyzed by unpaired Student's t-test for significant differences between the mean values of each group.

## 3 Results and discussion

For the comprehensive investigation of NPTNβ-regulated genes, we performed an RNA-seq-based analysis, and this was followed by the appearance of several genes with differing expressions between the GFP-expressed control A549 cells and their NPTNβ-overexpressed counterparts. We classified the altered genes into their disease categories (Fig. 1A), and the collected genes in the CANCER category (Fig. 1B) and in the other categories (Suppl. figS3Figs. S3A–F) are displayed as heat map lists. Regarding the genes on the CANCER list, we searched for each individual gene in the literature. We were curious about upregulated genes, especially the following six genes since they might play an important part in cancer aggressiveness.

(*1*) Fibroblast growth factor receptor 4 (FGFR4) has been shown to promote tumor invasion by coupling with FGF signaling [16]. (*2*) Insulin-like growth factor binding protein 2 (IGFBP2) acts as a potent oncogene to drive cancer metastasis [17] with an epithelial-mesenchymal transition (EMT) [18]. (*3*) Integrin subunit alpha 1 (ITGA1) and (*4*) integrin subunit beta 4 (ITGB4) are members of an integrin superfamily that acts to attach to various types of extracellular matrixes in various types of integrin complex between alpha and beta. It was reported that upregulated ITGA1 enhances the metastatic potential in pancreatic cancer cells [19], and that elevated ITGB4 promoted the metastasis of gastric cancer [20] and hepatocellular carcinoma [21]. (*5*) Kruppel-like factor 5 (KLF5) is a transcription factor that can promote cancer proliferation, migration, and invasion via the upregulation of its several targeted oncogenic genes [22,23]. Lastly, (*6*) solute carrier family 22 member 18 antisense (SLS22A18AS) is also interesting since the antisense blocks its sense gene (SLC22A18, which acts as a tumor suppressor) [24]. We thus speculated that the elevation of SLS22A18AS might enhance cancer aggressiveness through the prevention of tumor suppressive function(s) of its target sense.

The expression profiles of our six genes of interest were further confirmed by the quantitative-real time PCR analysis results (Fig. 1C). Of these six genes, we were particularly interested in SLC22A18AS because it showed the highest expression rate in elevation among the upregulated six genes, and there are only a few reports about its function.

**Fig. 2:**
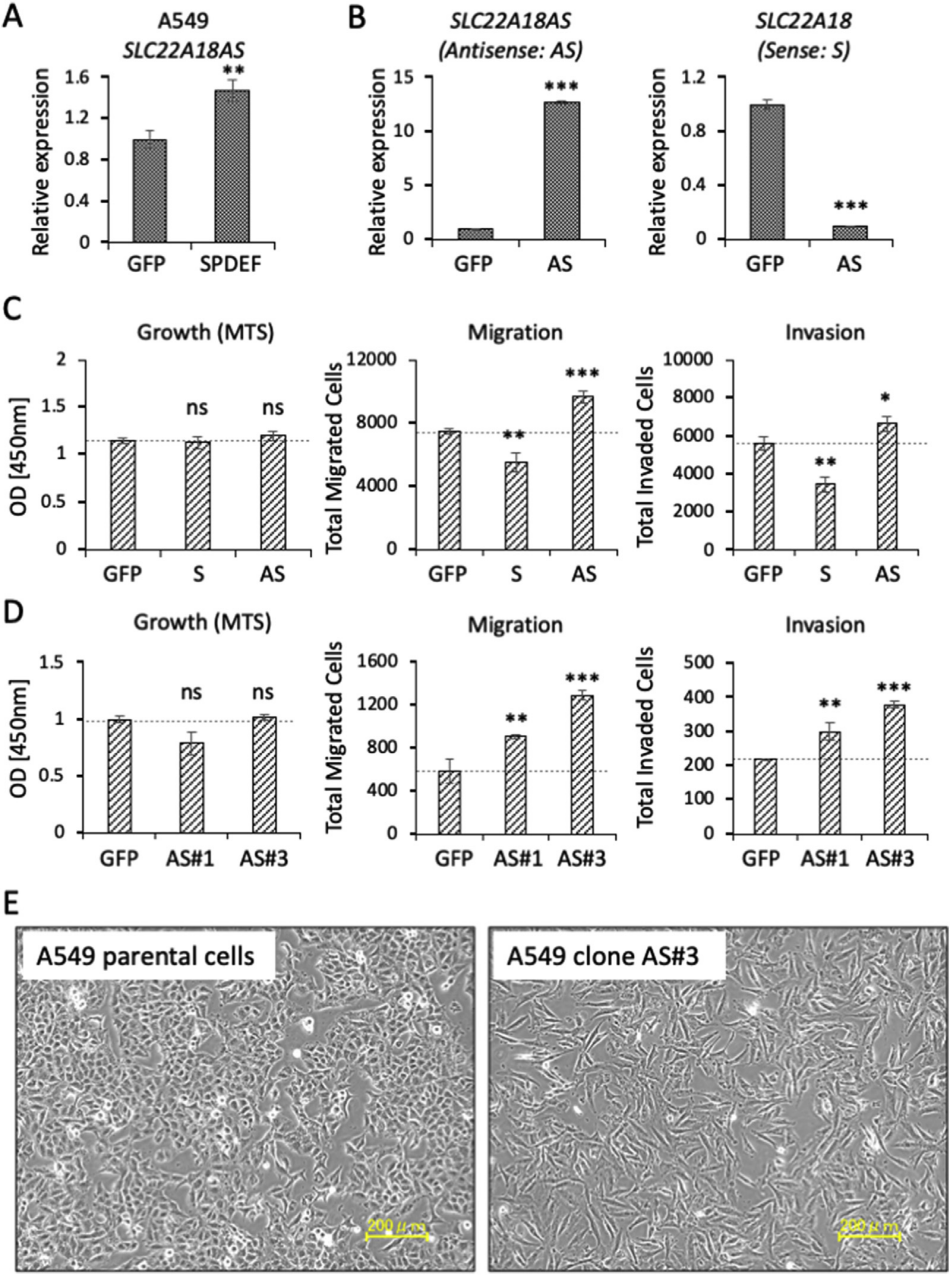
**The SLC22A18AS-mediated upregulation of cellular motility. A:** A quantitative real-time PCR analysis of *SLC22A18AS* gene was performed in the cells that were transiently overexpressed with either GFP or SPDEF. **B:** A quantitative real-time PCR analysis for *SLC22A18AS* (*Antisense: AS*) and *SLC22A18* (*Sense: S*) genes was performed in the cells that were transiently overexpressed with either GFP or SLC22A18AS (AS). **C:** A549 cells were transiently transfected with GFP as a control, SLC22A18 (S), or SLC22A18AS (AS) expression vector for 24 h. The cell growth of each sample was evaluated by an MTS assay (*left*). The migration (*middle*) and invasion (*right*) of A549 cells transfected with the indicated gene for 24 h were also assessed by the Boyden chamber method. The transfected A549 cells were placed in the top chamber and kept for 12 h. Migrated/invaded cells were quantified by cell counting in five non-overlapping fields at × 100 magnification and are presented as the average of three independent experiments. **D:** The stably expressed clones for the indicated genes (GFP or SLC22A18AS, which corresponds to clones AS#1 and AS#3) were assessed for growth (*left*), migration (*middle*), and invasion (*right*) according to the same methods as described in (**C**). Data are mean ± SD. *p<0.05, **p<0.01, ***p<0.001. **E:** Images of the indicated cells were obtained by phase-contrast microscopy. Scales: 200 μm.

Because SPDEF transcriptional factor is a key mediator of NPTNβ-triggered lung cancer dissemination, we first examined the relationship between SPDEF and SLC22A18AS expression. As shown in Fig. 2A, we observed that SPDEF has the ability to induce SLC22A18AS in A549 cells. However, this induction rate was not comparable to that caused by stable overexpression with NPTNβ (Fig. 1C), implying a certain contribution of another NPTNβ-mediated pathway that may orchestrate with SPDEF's functions to lead upregulation of SLC22A18AS in transcriptional expression (Suppl. Fig. S6C).

To further test the antisense function that exerts a degradation of its targeted-sense mRNA after complementary binding (like siRNA), we next transiently forced the expression of foreign SLC22A18AS in A549 cells. The expressed antisense in the transduced cells showed markedly higher expression than that in the control GFP-delivered cells (Fig. 2B). Under these conditions, we confirmed that the mRNA level of the intrinsic SLC22A18 gene was markedly reduced. This result indicates that SLC22A18AS works to bond with the complementary chain of its sense mRNA (SLC22A18) as the antisense trait, leading to a downregulation of SLC22A18.

We next examined the function(s) of SLC22A18AS coupled with the downregulation of SLC22A18 in cancer behaviors such as cell growth, migration, and invasion. Of interest, although the transient expression of both sense and antisense of SLC22A18 showed no major differences between them or when compared to that of the control GFP on the MTS-based growth evaluation, we observed that the antisense expression promoted either migration or invasion, whereas the sense expression highly attenuated these cellular behaviors (Fig. 2C). The upregulation of migration and invasion was also observed when we used the established A549 clones (AS#1 and #3), which showed high expressions of SLC22A18AS in a stable manner (Fig. 2D, *middle* and *right panels*).

The cell migration activity was further assessed by a more sophisticated assay, the real-time cell mobility assay using the device EZ-TAXIScan (ECI, Kawasaki, Japan), which provides multiple types of information about the migrating cells together with cell morphology, directionality, and velocity [[25], [26], [27]]. This additional analysis strengthened the former results, i.e., both of the AS clones showed markedly high activities in both the directionality and the velocity of migration compared to the activity of the control GFP clone (Suppl. Fig. S4A). The growth was not affected in the AS clones.

This was also confirmed by another cell counting method (Fig. 2D, *left panel*, Suppl. Fig. S4B). Both of the AS clones showed a slightly decreased trend in this assay. These results suggest that SLC22A18AS acts to enhance cancer migration and invasion processes but not growth, and the level of SLC22A18AS represent cancer dissemination through an inhibitory mechanism of its targeted sense mRNA.

**Fig. 3:**
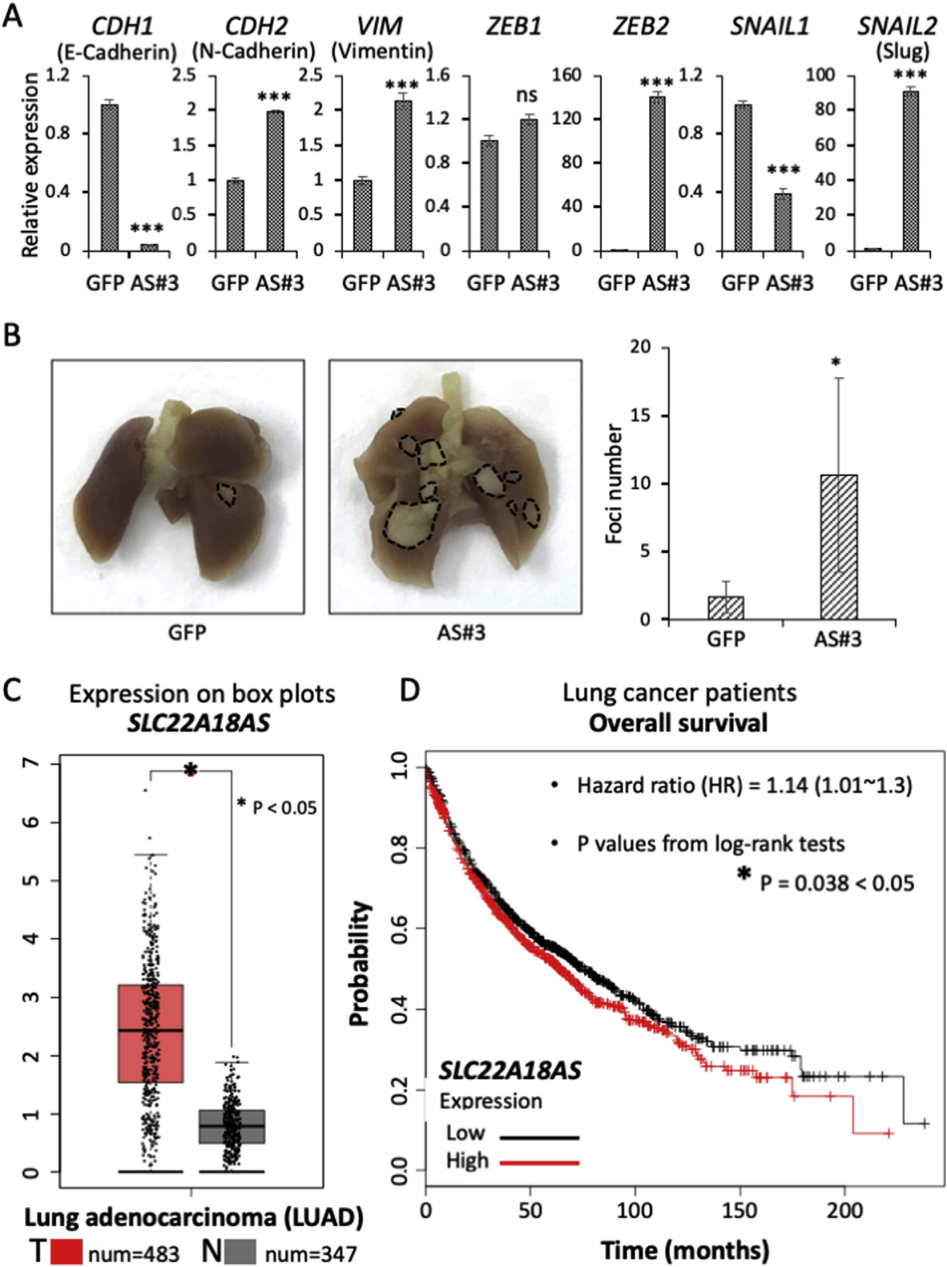
**SLC22A18AS-mediated EMT and dissemination and their relevance to survival suppression. A:** A quantitative real-time PCR analysis of the EMT marker genes was performed in the stably expressed clone, GFP, or AS#3, which corresponds to GFP or *SLC22A18AS* gene. **B:** A549 parental cells and an A549 cell-based stable trasnformant, AS#3 (each 5 × 10^5^ cells, with Matrigel) were injected into the lungs of mice and maintained for 1 month. *Left panel:* Representative photographs of the transplanted cell-derived tumors in the resected lungs. The visible tumors are encircled by *dotted lines* and quantified by counting the tumor foci of the resected lungs (*right panel*). **C:** Gene expression plots of *SLC22A18AS* from lung cancer specimens were obtained from a publicly available website (http://gepia.cancer-pku.cn/). **D:** Overall survival plots according to *SLC22A18AS* expression levels were obtained from a publicly available website (http://kmplot.com/analysis/). Data are mean ± SD. *p<0.05, ***p<0.001.

Because cancer dissemination is triggered by an acquisition of the EMT [28], we examined the possible linkage of SLC22A18AS and the expression of the EMT phenotype in lung cancer cells. Phase contrast microscopy revealed the appearance of morphological changes of the SLC22A18AS clone AS#3 from the epithelial shape to a mesenchymal fibroblast-like shape (Fig. 2E). This was not restricted to AS#3, as the other clone AS#1 showed shapes that were similar to those of AS#3 (data not shown). The morphological change toward the mesenchymal phenotype in clone AS#3 was confirmed by our evaluation of the cell-shape-based quantification (Suppl. Fig. S5) as well as the expression of several EMT markers (Fig. 3A).

A paper by Dey et al. [29] was of great help to us in conducting the morphological assessment with a detailed and accurate quantification. They used differential interference contrast (DIC) microscopy, which provides a pseudo-3D (three-dimensional) image of the cell. By learning their method and by using several other evaluation methods reviewed by Kasprowicz et al. [30], we were eventually able to take advantage of a simple enclosed method of cell examination that conveniently provides altered-cell shape information when combined with an ImageJ analysis. With this approach, we observed that the cell morphology of clone AS#3 was clearly changed compared to the cell morphology of the parental cells, i.e., it showed a mesenchymal-like shape with an extended area and extended major axis but not minor axis (Suppl. Fig. S5). In addition, as shown in Fig. 3A, the expression of the epithelial marker cadherin 1/E-cadherin (CDH1) was reduced and the expressions of the following mesenchymal markers were increased in the AS#3 clone compared to their expression in the control GFP clone: CDH1, vimentin (VIM), zinc finger E-box binding homeobox 2 (ZEB2), and snail family transcriptional repressor 2/slug (SNAIL2).

Regarding the altered EMT genes, ZEB2 and SNAIL2 displayed particularly high expression in the clone AS#3. This result is very interesting for us since it was demonstrated that coupled ZEB2/SNAIL2 signaling plays a crucial role in EMT acquisition and the subsequent disseminative metastasis of lung cancer [31]. There may be a close relationship between ZEB2/SNAIL2-mediated EMT and metastatic dissemination, since we observed herein that clone AS#3 showed many disseminated tumor foci compared to those from the parental A549 cells in the lung when we inoculated them simultaneously into a single site in individual mouse lungs, suggesting a highly disseminative activity of the clone (Fig. 3B).

Under the *in vivo* conditions of the mouse model, we assessed the EMT markers, ZEB1, ZEB2, SNAIL1 and SNAIL2, expression in the injected cancer-derived nodules collected from the dissected whole lung tissues (Suppl. Fig. S6A, *left*). It thus followed that AS#3-derived cancer nodules show markedly higher expressions of ZEB2 and SNAIL2 than those of the control GFP clone-derived cancer nodules (Suppl. Fig. S6A, *right*). On the other hand, ZEB1 showed no any appreciable alteration in expression between the two tumor specimens (GFP and AS#3) and SNAIL1 was not detected in the both specimens.

To determine whether SPDEF regulates ZEB2 and SNAL2, we examined ZEB2 and SNAIL2 again in the SPDEF knockdown model of clone A549-NPTNβ (a stable clone of A549 cells that shows persistent overexpression of NPTNβ). Under the siRNA-mediated reduced expression of SPDEF at an efficient level, we observed that the expression of ZEB2 was actually reduced, whereas SNAIL2 was not affected (Suppl. Fig. S6B). Taken together, these findings led us to consider that SPDEF mediates NPTNβ-triggered lung cancer dissemination through an SLC22A18AS-ZEB2 axis. However, the role of SPDEF is controversial since several studies indicated a cancer suppressive role of SPDEF in prostate cancer [32,33] and colorectal cancer [34,35] (Suppl. Fig. S6C). One possible explanation may arise different experimental settings and different cell types that involve one or more regulator molecules and the relevant pathways to regulate the expression and function of SPDEF. This complex topic provides a new direction for our research.

For the assessment of the clinical importance of SLC22A18AS in lung cancer, we next investigated the expression state of the gene and its relevance to patient survival. Publicly available data were used in these evaluations. The results of the expression analysis presented in box plots (http://gepia.cancer-pku.cn/) showed highly elevated expressions of SLC22A18AS in patient-derived lung adenocarcinoma specimens compared to the expression in healthy lung specimens (Fig. 3C). In addition, Kaplan-Meier survival plots (http://kmplot.com/analysis/) demonstrated that the patients with a high expression of SLC22A18AS had lower overall survival compared to the patients with a low expression (Fig. 3D). These results imply that the NPTNβ-mediated upregulation of SLC22A18AS is a novel risk factor for the induction of metastasis, leading to poorer survival in lung cancer patients.

In light of our results, it appears that the SLC22A18AS-induced disseminative activity that is concomitant with the EMT might be mediated by the prevention of its targeted sense SLC22A18. Thus, SLC22A18 seems to function as a tumor suppressor, as was reported [24]. However, it remains unknown how SLC22A18 suppresses the EMT and cellular migration and invasion. SLC22A18 is one type of membrane transporter, and it may regulate the uptake and pumping out of one or more important molecules associated with cancer metastasis and the EMT. The identification of such molecule(s) is part of our ongoing research.

It should also be determined whether SLC22A18AS has important target molecule(s) other than SLC22A18 that may unexpectedly affect cancer progression with the EMT and the subsequent cancer dissemination. However, we have not identified any reports about target molecules of SLC22A18AS. In addition, unlike the case for miRNA, it is too difficult to identify target molecules by searching for matched sequences on the basis of the SLC22A18AS sequence because of its great length. Further comprehensive analyses of gene expression using cancer cells that show up- or down-regulation of SLC22A18AS will help clarify whether new target molecules associated with cancer exist.

In conclusion, our data indicate that SLC22A18AS is a key molecule for the induction of the cancer EMT and the subsequent elevated cellular motility that is potentially linked to high metastatic risk (Suppl. Fig. S6C). The induction of the identified gene is regulated by the S100A8/A9-NPTNβ-SPDEF axis that we identified, which greatly contributes to lung cancer dissemination. However, SPDEF has contradictory functions in cancer progression: one is cancer dissemination and the other is cancer suppression. Our research has demonstrated that NPTNβ regulates the expression of many molecules that include not only SLC22A18AS but also previously reported cancer-relevant molecules whose functions are tightly associating with EMT progression such as FGFR4 [16], IGFBP2 [17,18], ITGA1 [19], ITGB4 [20,21], and KLF5 [22,23] (Fig. 1C). We thus contemplate that these molecules may affect the balance of SPDEF-mediated pathways, resulting in an enhancement of the cancer dissemination but not cancer suppression. We hope that our results will help establish a preclinical method to target this axis in order to further improve clinical responses and patient survival. In fact, our newly developed biologics that block the interaction between S100A8/A9 and NPTNβ (as well as other receptors; e.g., EMMPRIN, RAGE, MCAM, and ALCAM) have prevented the metastasis of melanoma and breast cancer [36,37].

## Funding

This research was supported in part by a grant from the 10.13039/501100001691JSPS KAKENHI (no. 17H03577 to M.S.) and by funds to M.S. from the 10.13039/501100004330Smoking Research Foundation, the Terumo Life Science Foundation, and the 10.13039/100007449Takeda Science Foundation.

## CRediT authorship contribution statement

**Karolina Bajkowska:** Data curation, Formal analysis, Investigation. **I. Wayan Sumardika:** Data curation, Formal analysis, Investigation, Methodology. **Nahoko Tomonobu:** Data curation, Formal analysis, Investigation, Methodology, Validation, Visualization. **Youyi Chen:** Formal analysis. **Ken-ichi Yamamoto:** Formal analysis. **Rie Kinoshita:** Formal analysis. **Hitoshi Murata:** Formal analysis. **Ni Luh Gede Yoni Komalasari:** Formal analysis. **Fan Jiang:** Formal analysis. **Akira Yamauchi:** Formal analysis. **I. Made Winarsa Ruma:** Formal analysis. **Carlos Ichiro Kasano-Camones:** Formal analysis. **Yusuke Inoue:** Formal analysis, Validation. **Masakiyo Sakaguchi:** Conceptualization, Funding acquisition, Methodology, Project administration, Resources, Supervision, Writing - original draft, Writing - review & editing.

## Declaration of competing interest

The authors declare that they have no conflicts of interest.
