# Supplementary material for: Neuroplastinβ-mediated upregulation of solute carrier family 22 member 18 antisense (SLC22A18AS) plays a crucial role in the epithelial-mesenchymal transition, leading to lung cancer cells' enhanced motility
Source: Biochem Biophys Rep. 2020 May 17;22:100768. doi: 10.1016/j.bbrep.2020.100768 (PMC7261704; doi:10.1016/j.bbrep.2020.100768)
Supplement: Multimedia component 1 [file mmc1.pptx]

## Slide 1
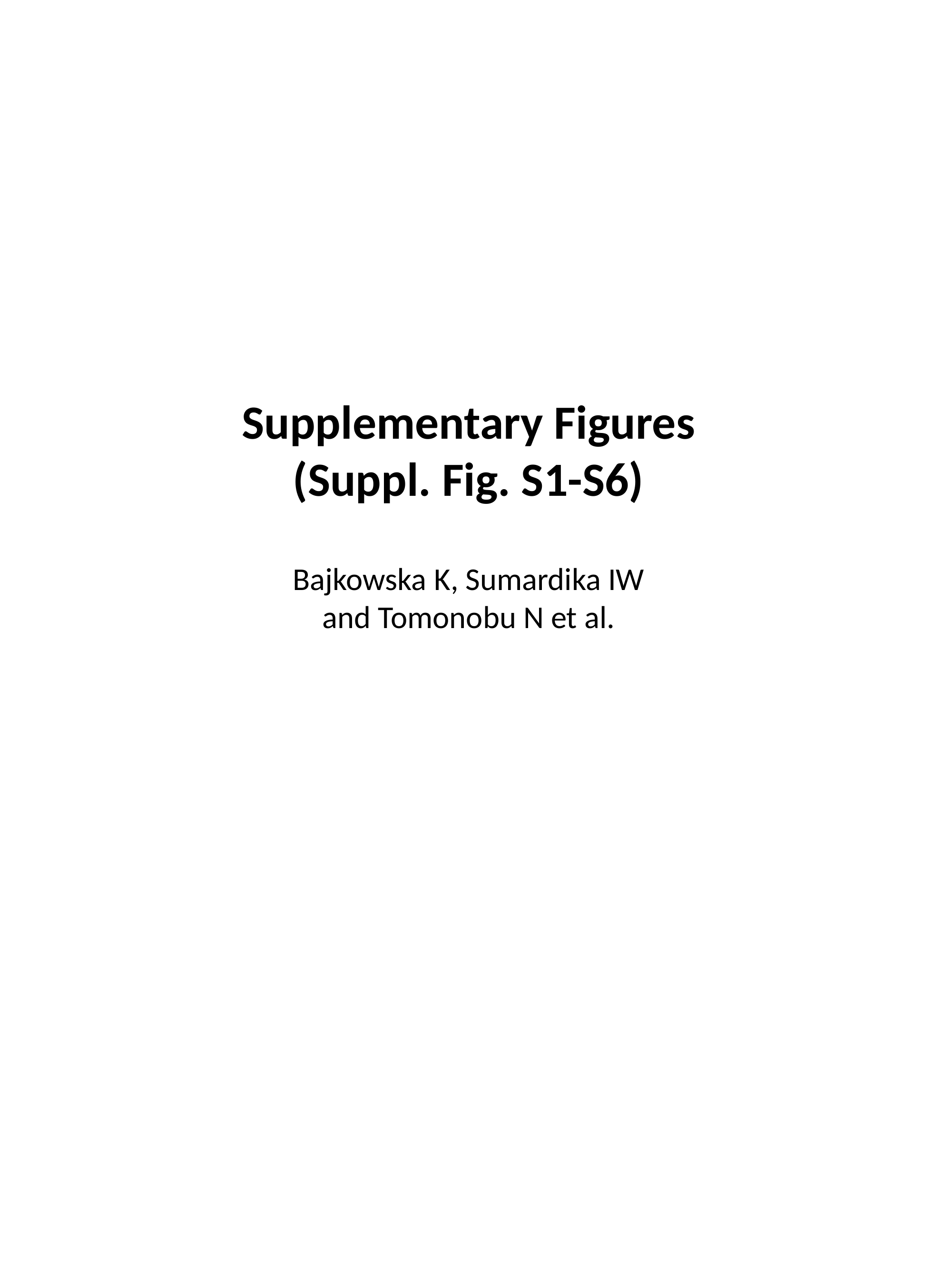

Supplementary Figures
(Suppl. Fig. S1-S6)
Bajkowska K, Sumardika IW and Tomonobu N et al.

## Slide 2
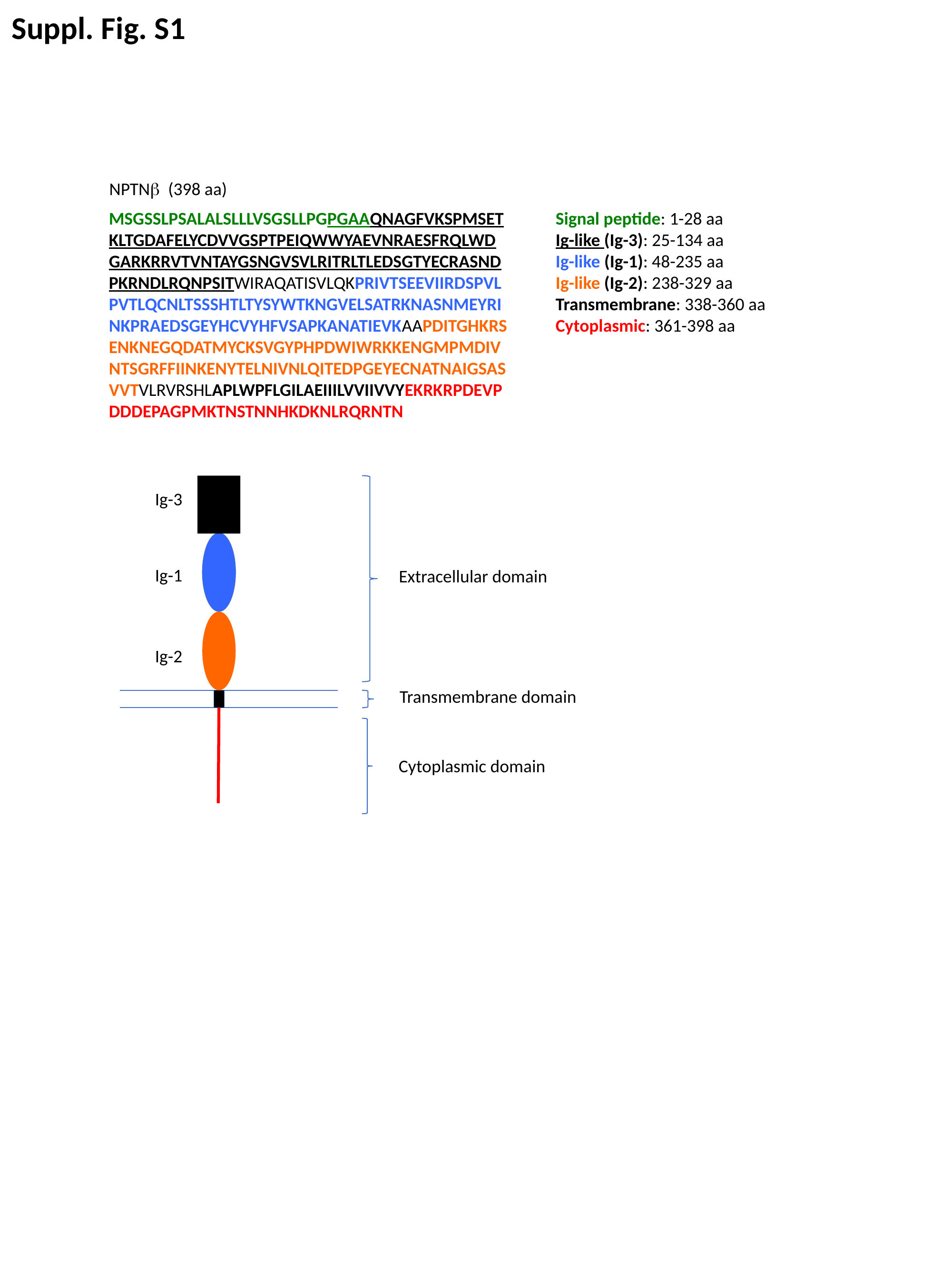

Suppl. Fig. S1
NPTNb
(398 aa)
MSGSSLPSALALSLLLVSGSLLPGPGAAQNAGFVKSPMSETKLTGDAFELYCDVVGSPTPEIQWWYAEVNRAESFRQLWDGARKRRVTVNTAYGSNGVSVLRITRLTLEDSGTYECRASNDPKRNDLRQNPSITWIRAQATISVLQKPRIVTSEEVIIRDSPVLPVTLQCNLTSSSHTLTYSYWTKNGVELSATRKNASNMEYRINKPRAEDSGEYHCVYHFVSAPKANATIEVKAAPDITGHKRSENKNEGQDATMYCKSVGYPHPDWIWRKKENGMPMDIVNTSGRFFIINKENYTELNIVNLQITEDPGEYECNATNAIGSASVVTVLRVRSHLAPLWPFLGILAEIIILVVIIVVYEKRKRPDEVPDDDEPAGPMKTNSTNNHKDKNLRQRNTN
Signal peptide: 1-28 aa
Ig-like (Ig-3): 25-134 aa
Ig-like (Ig-1): 48-235 aa
Ig-like (Ig-2): 238-329 aa
Transmembrane: 338-360 aa
Cytoplasmic: 361-398 aa
Ig-3
Ig-1
Ig-2
Extracellular domain
Transmembrane domain
Cytoplasmic domain

## Slide 3
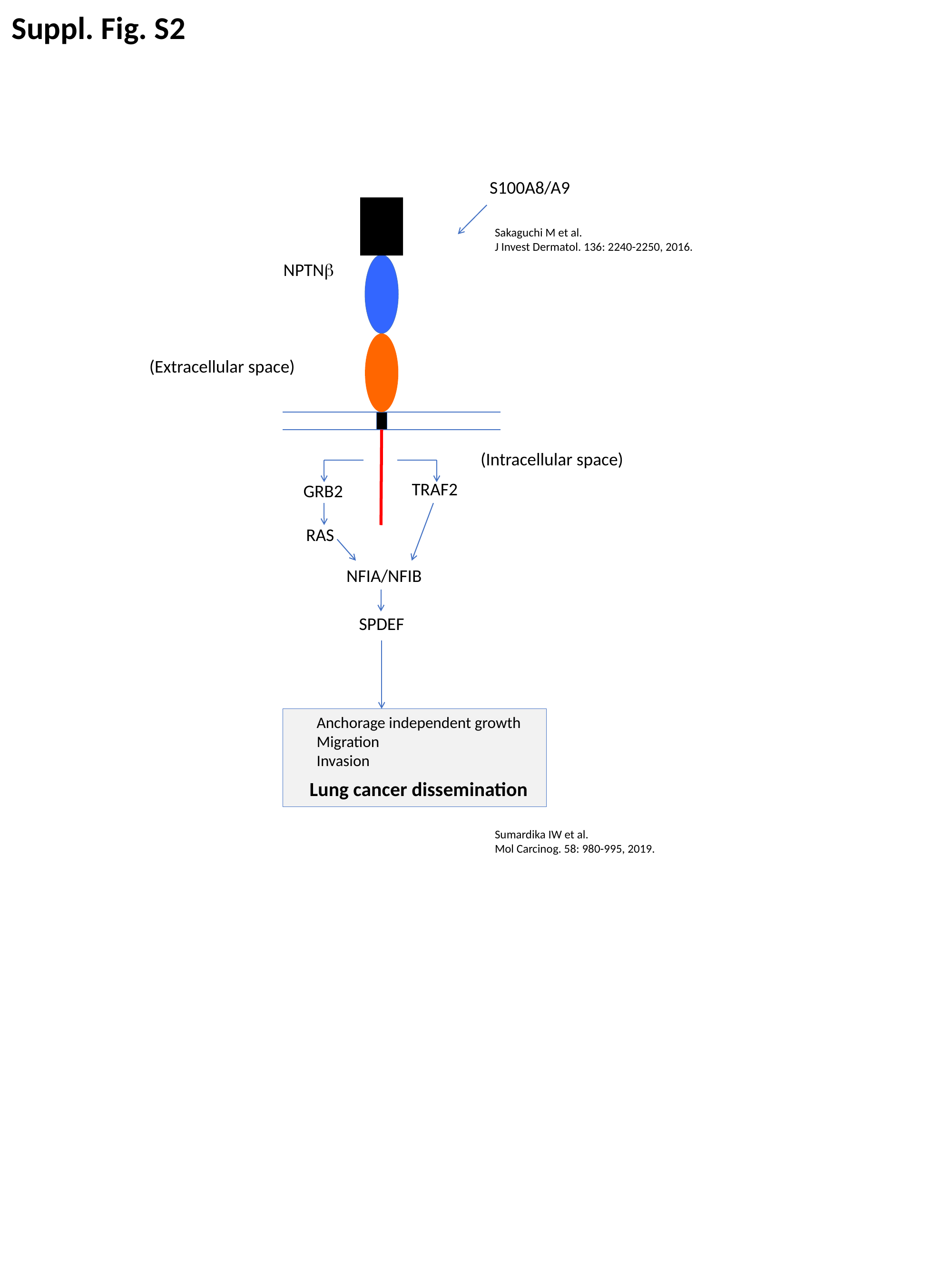

Suppl. Fig. S2
S100A8/A9
Sakaguchi M et al.
J Invest Dermatol. 136: 2240-2250, 2016.
NPTNb
(Extracellular space)
(Intracellular space)
TRAF2
GRB2
RAS
NFIA/NFIB
SPDEF
Anchorage independent growth
Migration
Invasion
Lung cancer dissemination
Sumardika IW et al.
Mol Carcinog. 58: 980-995, 2019.

## Slide 4
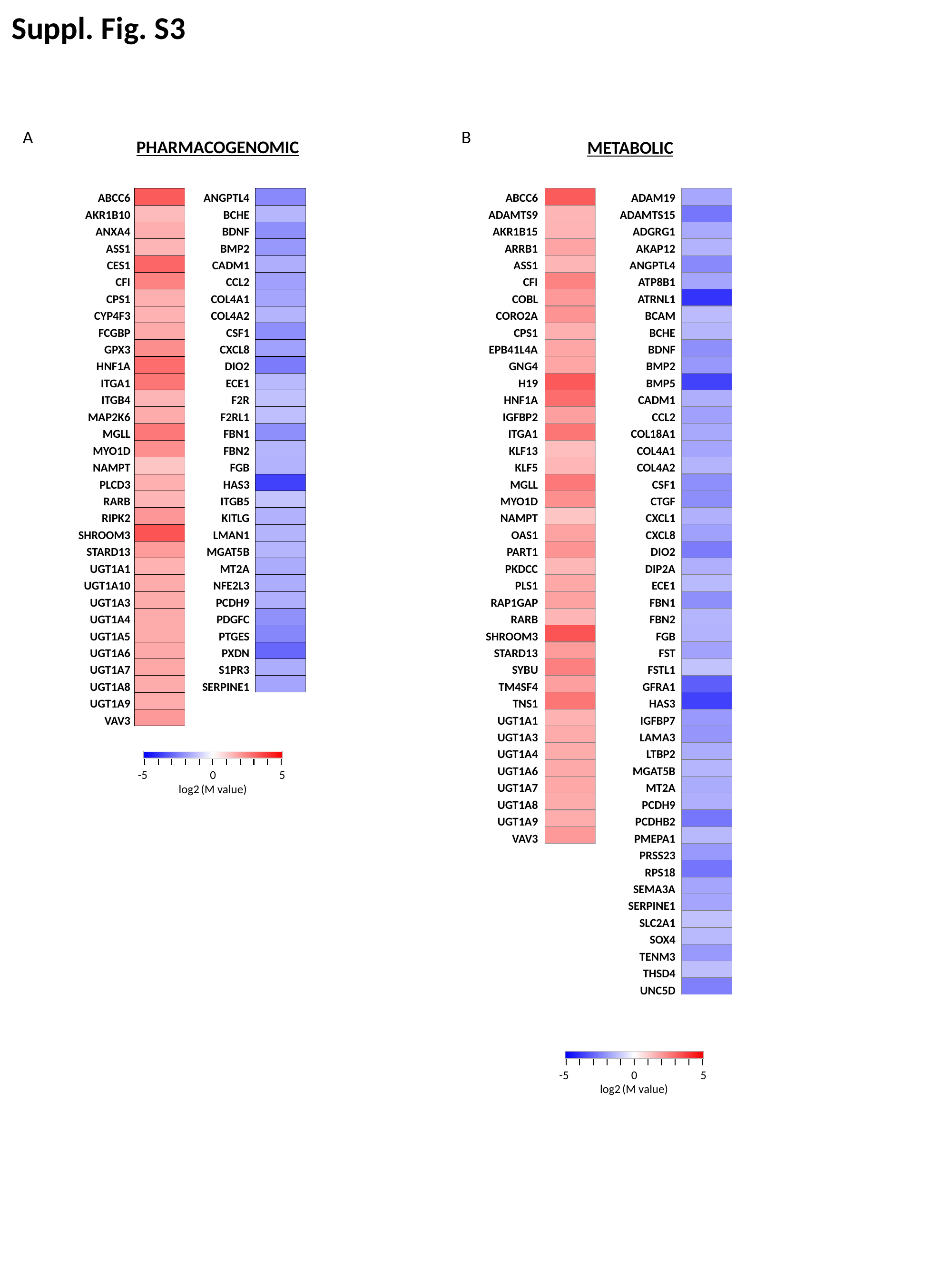

Suppl. Fig. S3
A
B
PHARMACOGENOMIC
METABOLIC
ABCC6
AKR1B10
ANXA4
ASS1
CES1
CFI
CPS1
CYP4F3
FCGBP
GPX3
HNF1A
ITGA1
ITGB4
MAP2K6
MGLL
MYO1D
NAMPT
PLCD3
RARB
RIPK2
SHROOM3
STARD13
UGT1A1
UGT1A10
UGT1A3
UGT1A4
UGT1A5
UGT1A6
UGT1A7
UGT1A8
UGT1A9
VAV3
ANGPTL4
BCHE
BDNF
BMP2
CADM1
CCL2
COL4A1
COL4A2
CSF1
CXCL8
DIO2
ECE1
F2R
F2RL1
FBN1
FBN2
FGB
HAS3
ITGB5
KITLG
LMAN1
MGAT5B
MT2A
NFE2L3
PCDH9
PDGFC
PTGES
PXDN
S1PR3
SERPINE1
ABCC6
ADAMTS9
AKR1B15
ARRB1
ASS1
CFI
COBL
CORO2A
CPS1
EPB41L4A
GNG4
H19
HNF1A
IGFBP2
ITGA1
KLF13
KLF5
MGLL
MYO1D
NAMPT
OAS1
PART1
PKDCC
PLS1
RAP1GAP
RARB
SHROOM3
STARD13
SYBU
TM4SF4
TNS1
UGT1A1
UGT1A3
UGT1A4
UGT1A6
UGT1A7
UGT1A8
UGT1A9
VAV3
ADAM19
ADAMTS15
ADGRG1
AKAP12
ANGPTL4
ATP8B1
ATRNL1
BCAM
BCHE
BDNF
BMP2
BMP5
CADM1
CCL2
COL18A1
COL4A1
COL4A2
CSF1
CTGF
CXCL1
CXCL8
DIO2
DIP2A
ECE1
FBN1
FBN2
FGB
FST
FSTL1
GFRA1
HAS3
IGFBP7
LAMA3
LTBP2
MGAT5B
MT2A
PCDH9
PCDHB2
PMEPA1
PRSS23
RPS18
SEMA3A
SERPINE1
SLC2A1
SOX4
TENM3
THSD4
UNC5D
-5
0
5
log2 (M value)
-5
0
5
log2 (M value)

## Slide 5
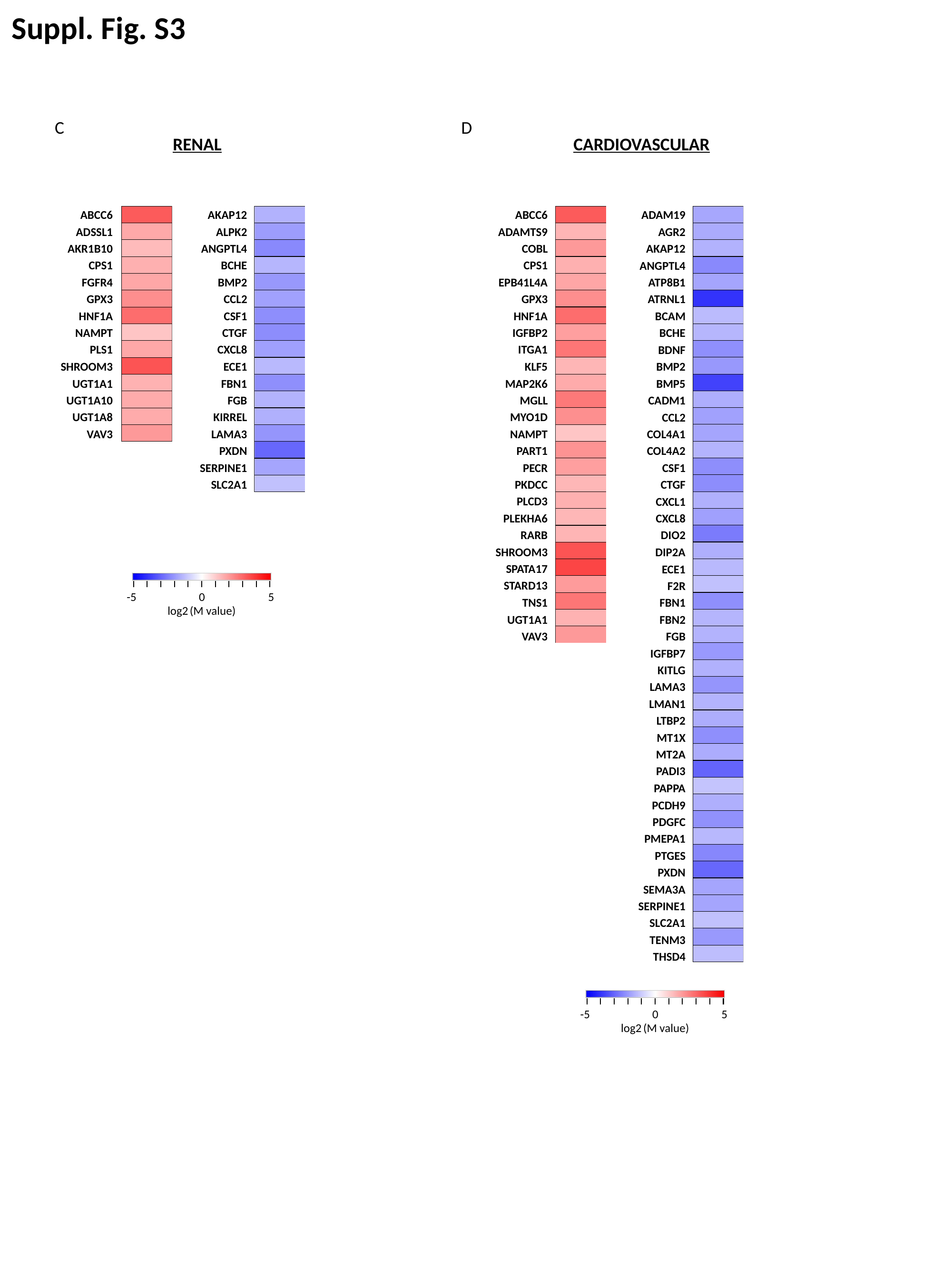

Suppl. Fig. S3
C
D
RENAL
CARDIOVASCULAR
ABCC6
ADSSL1
AKR1B10
CPS1
FGFR4
GPX3
HNF1A
NAMPT
PLS1
SHROOM3
UGT1A1
UGT1A10
UGT1A8
VAV3
AKAP12
ALPK2
ANGPTL4
BCHE
BMP2
CCL2
CSF1
CTGF
CXCL8
ECE1
FBN1
FGB
KIRREL
LAMA3
PXDN
SERPINE1
SLC2A1
ABCC6
ADAMTS9
COBL
CPS1
EPB41L4A
GPX3
HNF1A
IGFBP2
ITGA1
KLF5
MAP2K6
MGLL
MYO1D
NAMPT
PART1
PECR
PKDCC
PLCD3
PLEKHA6
RARB
SHROOM3
SPATA17
STARD13
TNS1
UGT1A1
VAV3
ADAM19
AGR2
AKAP12
ANGPTL4
ATP8B1
ATRNL1
BCAM
BCHE
BDNF
BMP2
BMP5
CADM1
CCL2
COL4A1
COL4A2
CSF1
CTGF
CXCL1
CXCL8
DIO2
DIP2A
ECE1
F2R
FBN1
FBN2
FGB
IGFBP7
KITLG
LAMA3
LMAN1
LTBP2
MT1X
MT2A
PADI3
PAPPA
PCDH9
PDGFC
PMEPA1
PTGES
PXDN
SEMA3A
SERPINE1
SLC2A1
TENM3
THSD4
-5
0
5
log2 (M value)
-5
0
5
log2 (M value)

## Slide 6
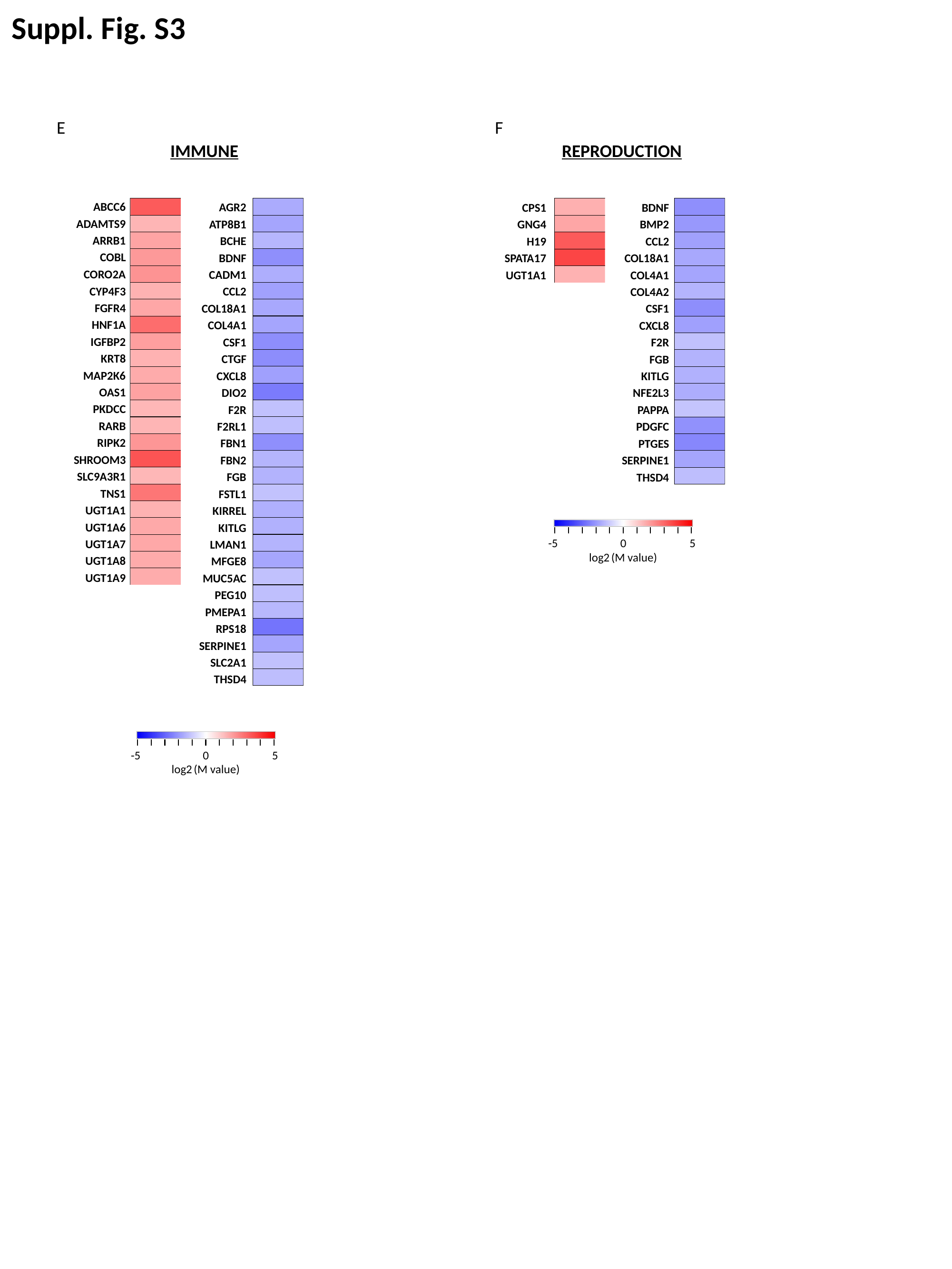

Suppl. Fig. S3
E
F
IMMUNE
REPRODUCTION
ABCC6
ADAMTS9
ARRB1
COBL
CORO2A
CYP4F3
FGFR4
HNF1A
IGFBP2
KRT8
MAP2K6
OAS1
PKDCC
RARB
RIPK2
SHROOM3
SLC9A3R1
TNS1
UGT1A1
UGT1A6
UGT1A7
UGT1A8
UGT1A9
AGR2
ATP8B1
BCHE
BDNF
CADM1
CCL2
COL18A1
COL4A1
CSF1
CTGF
CXCL8
DIO2
F2R
F2RL1
FBN1
FBN2
FGB
FSTL1
KIRREL
KITLG
LMAN1
MFGE8
MUC5AC
PEG10
PMEPA1
RPS18
SERPINE1
SLC2A1
THSD4
CPS1
GNG4
H19
SPATA17
UGT1A1
BDNF
BMP2
CCL2
COL18A1
COL4A1
COL4A2
CSF1
CXCL8
F2R
FGB
KITLG
NFE2L3
PAPPA
PDGFC
PTGES
SERPINE1
THSD4
-5
0
5
log2 (M value)
-5
0
5
log2 (M value)

## Slide 7
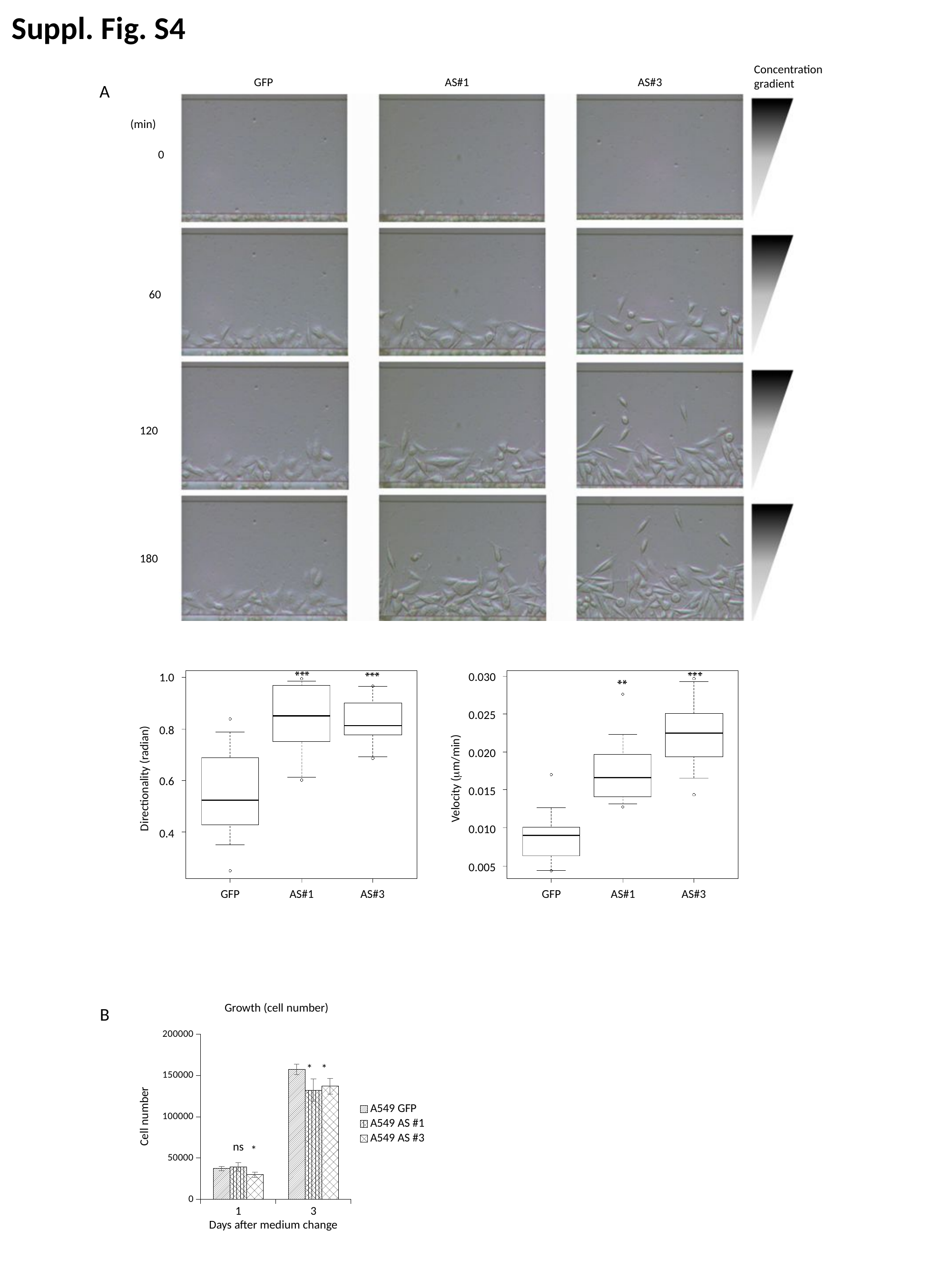

Suppl. Fig. S4
Concentration
gradient
GFP
AS#1
AS#3
(min)
0
60
120
180
A
1.0
0.8
Directionality (radian)
0.6
0.4
GFP
AS#1
AS#3
0.030
0.025
0.020
Velocity (mm/min)
0.015
0.010
0.005
GFP
AS#1
AS#3
Growth (cell number)
B
### Chart
| Category | A549 GFP | A549 AS #1 | A549 AS #3 |
|---|---|---|---|
| 1.0 | 37166.66666666658 | 39166.66666666658 | 29833.33333333331 |
| 3.0 | 157500.0 | 132250.0 | 137000.0 |*
*
Cell number
ns
*
Days after medium change

## Slide 8
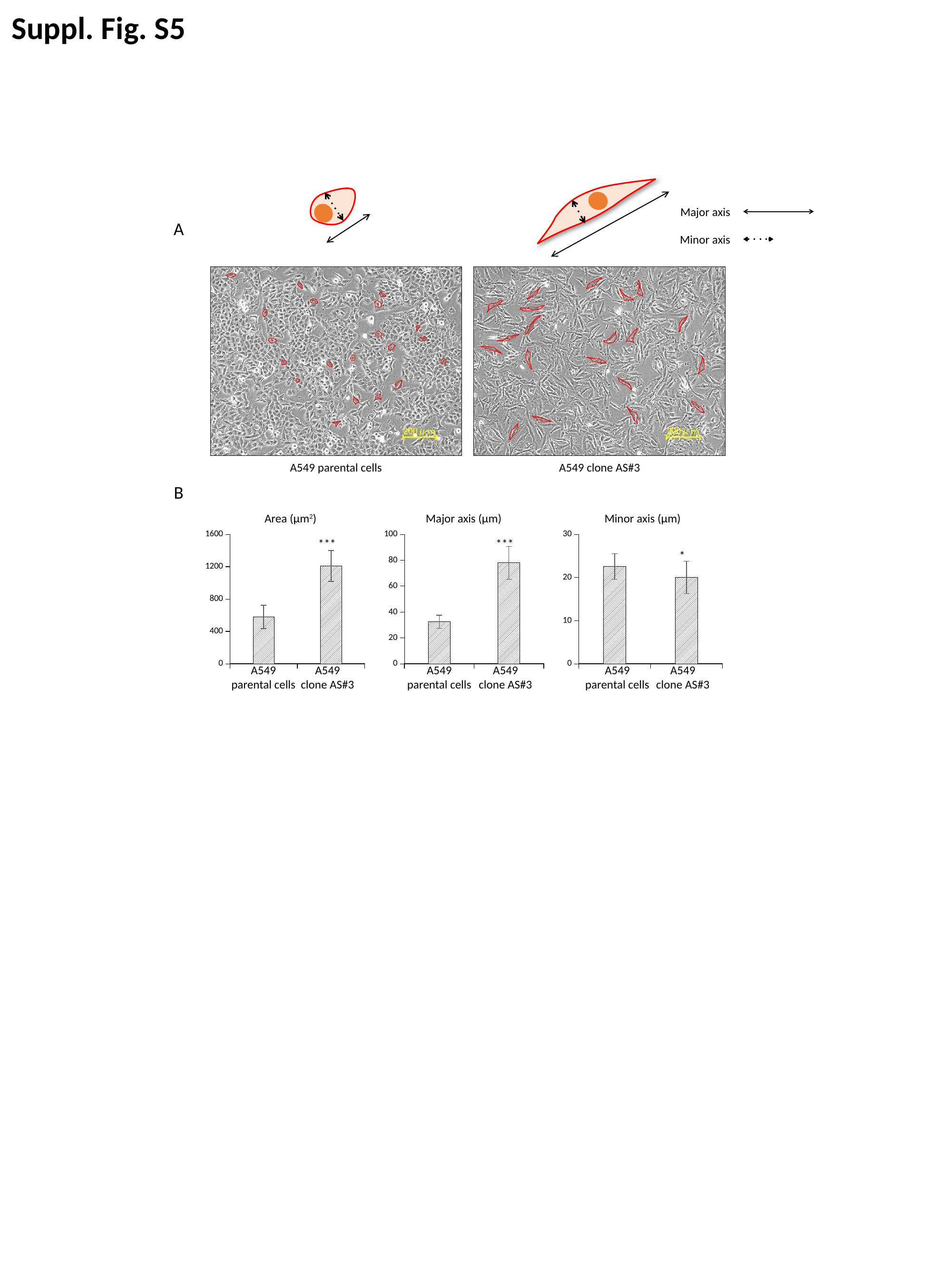

Suppl. Fig. S5
Major axis
A
Minor axis
A549 parental cells
A549 clone AS#3
B
Area (μm2)
Major axis (μm)
Minor axis (μm)
### Chart
| Category | |
|---|---|
| WT | 579.4270833333334 |
| AS#3 | 1208.767361111111 |
### Chart
| Category | |
|---|---|
| WT | 32.44791666666642 |
| AS#3 | 78.00385416666641 |
### Chart
| Category | |
|---|---|
| WT | 22.54864583333315 |
| AS#3 | 20.04395833333333 |***
***
*
A549
parental cells
A549
clone AS#3
A549
parental cells
A549
clone AS#3
A549
parental cells
A549
clone AS#3

## Slide 9
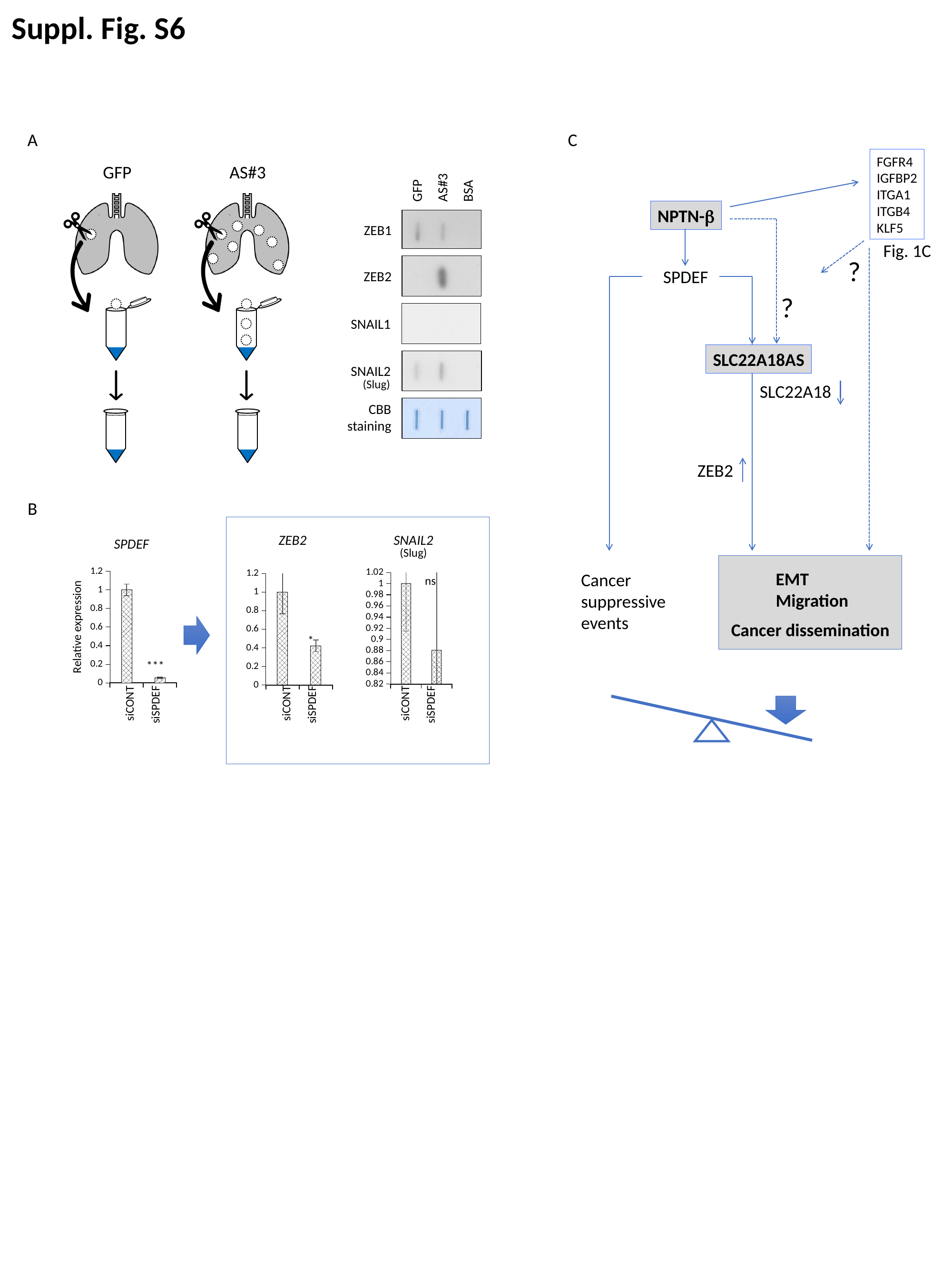

Suppl. Fig. S6
A
C
FGFR4
IGFBP2
ITGA1
ITGB4
KLF5
GFP
AS#3
AS#3
GFP
BSA
NPTN-b
ZEB1
ZEB2
SNAIL1
SNAIL2
(Slug)
CBB
staining
Fig. 1C
?
SPDEF
?
SLC22A18AS
SLC22A18
ZEB2
B
ZEB2
SNAIL2
SPDEF
(Slug)
### Chart
| Category | |
|---|---|
| siCONT | 1.0 |
| siSPDEF | 0.0550283289859653 |Relative expression
***
siCONT
siSPDEF
EMT
Migration
Cancer suppressive events
### Chart
| Category | |
|---|---|
| siCONT | 1.0 |
| siSPDEF | 0.880861064061071 |
### Chart
| Category | |
|---|---|
| siCONT | 1.0 |
| siSPDEF | 0.421387335429053 |ns
Cancer dissemination
*
siCONT
siSPDEF
siCONT
siSPDEF
